# Supplementary material for: bio2Byte Tools deployment as a Python package and Galaxy tool to predict protein biophysical properties
Source: Bioinformatics. 2024 Sep 6;40(9):btae543. doi: 10.1093/bioinformatics/btae543 (PMC11873786; doi:10.1093/bioinformatics/btae543)
Supplement: btae543_Supplementary_Data [file btae543_supplementary_data.pdf]

# Supplementary Information for "bio2Byte Tools deployment as a Python package and Galaxy tool to predict protein biophysical properties"

Jose Gavalda-Garcia, Adrián Díaz, Wim Vranken

September 3, 2024

## Supplementary information

Supplementary Table 1: **Tools description.** Simple description of every tool contained in bio2Byte tools.

| Predictor | Usage |
|---|---|
| DynaMine | Fast predictor of protein backbone dynamics using only sequence information as input. The current version also predicts side-chain dynamics and secondary structure predictors using the same principle. |
| DisoMine | Predicts protein disorder with recurrent neural networks not directly from the amino acid sequence, but instead from more generic predictions of key biophysical properties, here protein dynamics, secondary structure and early folding. |
| EfoldMine | Predicts from the primary amino acid sequence of a protein, which amino acids are likely involved in early folding events. |
| AgMata | Single-sequence based predictor of protein regions that are likely to cause beta-aggregation. |
| PSPer | PSP (Phase Separating Protein) predicts whether a protein is likely to phase-separate with a particular mechanism involving RNA interacts (FUS-like proteins). It will highlight protein regions that are involved mechanistically, and provide an overall score. |
| ShiftCrypt | Auto-encoding NMR chemical shifts from their native vector space to a residue-level biophysical index. |

Supplementary Table 2: **Overview of internal tool dependencies.** The predictors contained in this software suite often require the execution of other predictors in our suite. The optimisation process is explained in the main text and the list of dependencies is described in this table.

| Tool | Dependencies |
|---|---|
| DynaMine | None |
| EFoldMine | DynaMine |
| DisoMine | DynaMine, EFoldMine |
| AGmata | DynaMine, EFoldMine |
| PSPer | DynaMine, EFoldMine, DisoMine |
| ShiftCrypt | None |

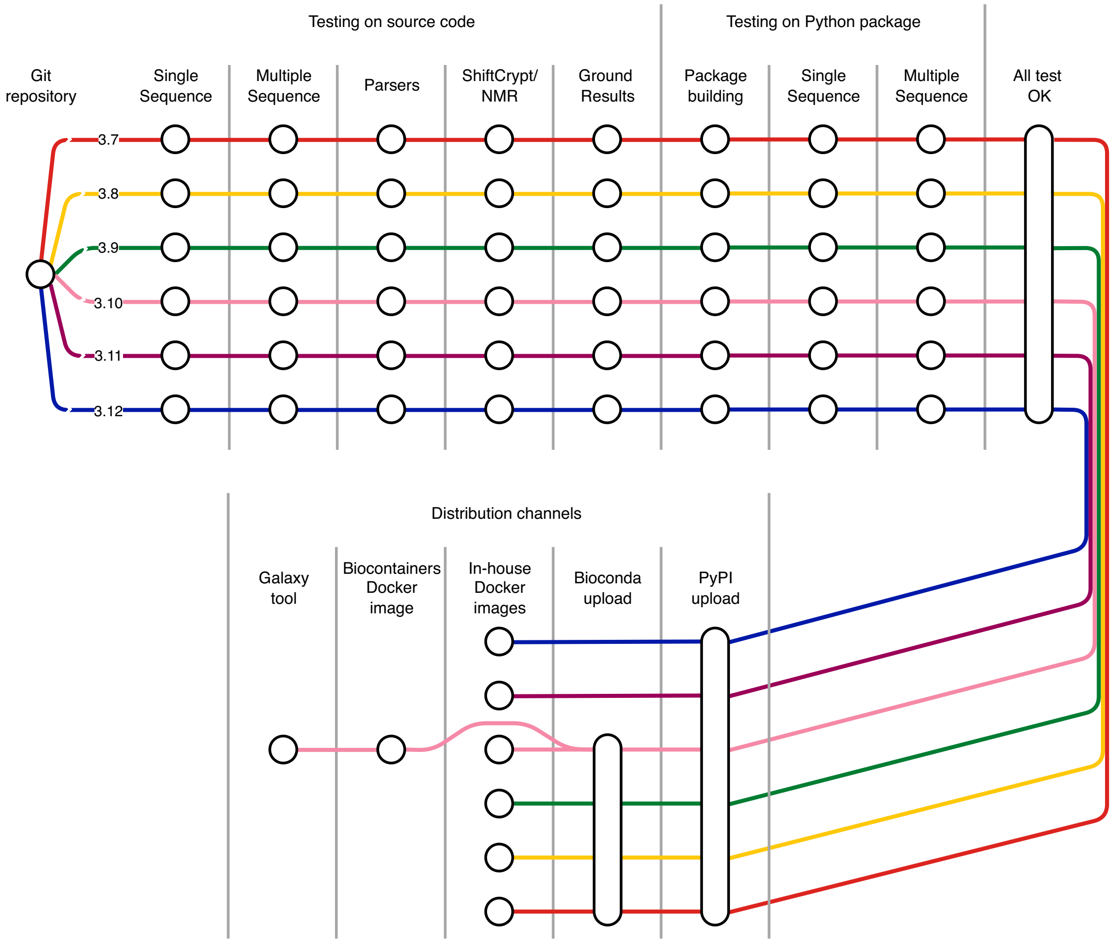


Supplementary Fig. 1: **Pytest process diagram.** The package testing process starts from a single Git repository, which then creates an environment per Python version from 3.7 to 3.12. For each environment, a series of tests on the source code are executed, namely the execution of single sequence, multiple sequence, parsers, and ShiftCrypt/NMR modules. Then the outputs get compared to ground results values and assessed for differences beyond float point errors (sum of absolute differences across all predicted metrics and residues, proteins and residues smaller than 0.001). A python package is then created and single sequence and multiple sequence modules are newly tested. If all tests across all Python versions are satisfactory, the python package will be uploaded to PyPI and to Bioconda. Then, an array of Docker images are created and uploaded to Docker Hub, following the settings in Supplementary Table 3. Finally, Bioconda automatically deposits a docker container with the Python 3.10 version of the package in Biocontainers, which can then be deployed into Galaxy.

Supplementary Table 3: **Python Versions and Distribution Sources for all the manually generated Docker images.** For each new release of bio2Byte Tools, the following set of Docker images are generated and published on `https://hub.docker.com/r/bio2byte/b2btools` for additional control over the version of Python and the provenance of the dependencies.

| **Python Version** | **Source** | **Description** |
|---|---|---|
| 3.7 | PyPI | Generated from PyPI distributions using Python 3.7. (Link to image) |
| 3.7 | Conda | Generated from Conda distributions using Python 3.7. (Link to image) |
| 3.7 | System | Generated from System packages using Python 3.7. (Link to image) |
| 3.8 | PyPI | Generated from PyPI distributions using Python 3.8. (Link to image) |
| 3.8 | Conda | Generated from Conda distributions using Python 3.8. (Link to image) |
| 3.8 | System | Generated from System packages using Python 3.8. (Link to image) |
| 3.9 | PyPI | Generated from PyPI distributions using Python 3.9. (Link to image) |
| 3.9 | Conda | Generated from Conda distributions using Python 3.9. (Link to image) |
| 3.9 | System | Generated from System packages using Python 3.9. (Link to image) |
| 3.10 | PyPI | Generated from PyPI distributions using Python 3.10. (Link to image) |
| 3.10 | Conda | Generated from Conda distributions using Python 3.10. (Link to image) |
| 3.10 | System | Generated from System packages using Python 3.10. (Link to image) |
| 3.11 | PyPI | Generated from PyPI distributions using Python 3.11. (Link to image) |
| 3.11 | Conda | Generated from Conda distributions using Python 3.11. (Link to image) |
| 3.11 | System | Generated from System packages using Python 3.11. (Link to image) |
| 3.12 | PyPI | Generated from PyPI distributions using Python 3.12. (Link to image) |
| 3.12 | Conda | Generated from Conda distributions using Python 3.12. (Link to image) |
| 3.12 | System | Generated from System packages using Python 3.12. (Link to image) |
